# Ion-Exchanged Beta-Based Cobalt Catalyst for Efficient Degradation of Aqueous Dye Acid Orange II

**DOI:** 10.3390/nano15211630

**Published:** 2025-10-26

**Authors:** En Fu, Xiang Liao, Chun He, Shaodan Xu, Huanxuan Li

**Affiliations:** College of Materials & Environmental Engineering, Hangzhou Dianzi University, No. 1158, Second Avenue, Xiasha Higher Education Zone, Hangzhou 310018, China

**Keywords:** Co/Beta catalyst, zeolite, ion-exchange, dye degradation

## Abstract

A highly active Co/Beta catalyst was prepared via ion-exchange method, in which sodium cations in the beta zeolite framework were replaced by cobalt ions using an aqueous cobalt nitrate solution. Based on XRD, SEM, TEM, XPS, and nitrogen adsorption–desorption analyses, it was confirmed that cobalt species successfully took the place of sodium ions in beta zeolite, while the cobalt species diffused with a uniform dispersion. Strong electronic coupling between cobalt species and zeolite framework oxygen stabilizes Co^2+^ sites in the material. The catalysts perform high efficiency in dye Acid Orange II (AO7) degradation reactions, which gives more than 99.5% removal efficiency at room temperature and initial pH within 10 min under low catalyst dosage. The advantages of the Co/Beta catalyst are reasonably attributed to its maximized metal−zeolite synergistic efficiency.

## 1. Introduction

Aqueous dye pollutants, such as acid orange 2, which has been widely used as a chemical in the dyeing and printing of silk, paper, and wool, have aroused extensive concern all over the world due to its persistence, accumulation, non-degradability, chronic toxicity, and carcinogenicity in the environment [1,2,3,4,5]. Thus, wastewater containing azo synthetic dyes has become one of the biggest challenges in water environmental safety. Effective remediation of synthetic dye contaminants is critical to mitigate aquatic ecosystem degradation and minimize toxicological hazards to human and ecological receptors. For the last decades, many traditional methods have been investigated for removing organic pollutants from polluted water, such as biological treatment [4,6,7,8], chemical treatment, and physical adsorption [6,9,10,11,12,13,14]. However, conventional biological treatment systems exhibit limited efficacy against recalcitrant compounds with molecular masses ranging from several thousand to tens of thousands. Although physical adsorption can also play a role in purifying water quality, pollutants are only transferred from water to adsorbents but not decomposed or completely eliminated. Among them, advanced oxidation processes (AOPs) represent a frontier technology for wastewater remediation, enabling direct contaminant mineralization or catalytic bio-susceptibility enhancement via radical-mediated oxidation, with exceptional efficacy against trace-level pollutants [15,16,17,18,19,20]. Therefore, AOPs boast extensive application prospects and high research value. Consequently, much more attention has been attracted from research and industrial fields.

Owing to their excellent catalytic performance, selectivity, and stability, metal catalysts based on zeolites have been widely explored and studied, which have been applied in various important reactions, such as Fischer Tropsch synthesis [21,22,23], propane dehydrogenation [24,25,26], pollutant degradations [27,28,29,30] and other important industrial catalytic reactions [31,32,33,34]. In the field of catalytic degradation of dye pollutants, catalysts are commonly synthesized via incipient wetness impregnation owing to their simplicity and broad applicability. However, the impregnation method has inherent limitations. The metal loading is restricted by both the pore volume of the molecular sieve and the solubility of the metal salt precursor. Moreover, the metal species at high loading levels tend to agglomerate and form large-sized ones, which is detrimental to the catalytic activity and stability. For the peroxymonosulfate (PMS) activation, the Co- or Fe-containing metal-organic frameworks (MOFs) have been extensively studied and are regarded as highly efficient catalysts [35,36,37]. It is worth noting that the MOFs with organic linkages might suffer from insufficient stability during the radical-rich conditions, limiting their wide applications. In addition, the cobalt, cobalt-iron nanocomposite, and Co (3)–acetate complexes have been used for the reaction, but the efficiency still needs further improvement [38,39]. Compared with these species, the zeolite materials could efficiently disperse the metal species and benefit the catalysis [40].

In this work, we employed the Beta zeolite with 12–MR micropores that exhibited higher activity with enhanced metal dispersion within the zeolite cages. The study employed an ion-exchange route to obtain the Co/Beta catalyst. The synthesis involved substituting the Na^+^ ions in beta zeolites with Co^2+^ through treatment with an aqueous cobalt nitrate solution. To assess the physical and chemical properties of catalysts, various characterization methods were adopted, and the performance in the degradation of AO7 was evaluated. The success was realized by its enhanced electrostatic interaction between these Co^2+^ ions and the oxygen atoms in the framework.

## 2. Experimental Section

### 2.1. Sample Preparation

***Materials.*** All reagents were of analytical grade and used as purchased without further purification.

***Synthesis of Beta zeolite.*** In a typical run, Beta zeolite was synthesized using tetraethylammonium hydroxide (TEAOH) as the template agent. As a typical run, NaAlO_2_, NaOH, TEAOH, and fumed SiO_2_ were mixed at room temperature and stirred until they formed a homogeneous gel with a nominal molar composition: 30SiO_2_: 1Al_2_O_3_: 1.5Na_2_O: 14TEAOH: 600H_2_O. Hydrothermal treatment of the obtained gel was performed at 140 °C for 48 h within a Teflon-lined autoclave. The autoclave was cooled to room temperature after the crystallization step. The solid product was recovered by centrifugation, washed several times with deionized water, and dried overnight at 120 °C, followed by calcination at 550 °C for 4 h in a flow of air. The NaBeta zeolite with an atomic Si/Al ratio of 15 was finally obtained.

***Synthesis of Co/Beta.*** As a typical experimental procedure, 200 mg of NaBeta was mixed with 350 mL of an aqueous solution containing 5.0 mg of cobalt nitrate hexahydrate. Continuous stirring of the mixture was carried out at 80 °C for a duration of 12 h, then filtered and dried. The other samples with different cobalt loading contents were synthesized following the same procedures except for the different initial amounts of cobalt nitrate (10.0 mg, 20.0 mg, 25.0 mg, and 30.0 mg). The catalysts were finally obtained and denoted as Co/Beta-x% (x was the actual cobalt content after ion-exchange, as determined by ICP-OES). Other Co/zeolite catalysts were synthesized under the same conditions, except for replacing NaBeta molecular sieves with NaY or NaZSM-5.

### 2.2. Sample Characterization

The powder X-ray diffraction analysis was performed on a Rigaku DMAX-2600 system utilizing Cu Kα radiation (λ = 0.1542 nm). XPS measurements were conducted using a Thermo Scientific™ Nexsa spectrometer, where the binding energies were calibrated against the C 1s peak (284.8 eV). The concentration of metals was quantified by inductively coupled plasma spectroscopy (ICP-OES, Agilent 5100). Scanning electron microscopy (SEM) and elemental mapping experiments were performed with Thermo Scientific Apreo 2C. HAADF-STEM images, along with elemental mapping, were recorded on the Thermo Fisher Talos F200S. The element scanning adopts the Super X EDS system for detection. For HAADF-STEM images and elemental mapping, samples floating on water or other solvents were gathered with a copper mesh equipped with a polymer microgrid. Nitrogen sorption measurements (BET) were performed with a Micromeritics ASAP Tristar device, following a degassing step of the samples at 300 °C under vacuum for 6 h.

### 2.3. Catalytic Tests

In conical flasks containing 30 mL of a 0.2 mM solution of AO7, batch tests were carried out at room temperature (25 ± 2 °C) using the initial pH of the solutions. In each experiment, a specified amount of catalyst (10 mg) was introduced into the reaction vessel. The reaction was then triggered by the addition of a certain quantity of PMS to the mixture. The solution was stirred at a water bath shaker (SHZ-C, Shanghai, China). For a given time, 2 mL of solution were periodically withdrawn with a syringe and filtered through a polyether sulfone (PES) filter. The concentration of AO7 was measured using a UV-Vis spectrometer at a wavelength of 486 nm. Each catalytic measurement was repeated three times, with very minor error bars, noting the excellent reproducibility of the catalytic tests. The influences of Co contents, reaction temperature, solution pH, and PMS loading on the degradation efficiency were investigated. Desired pH levels were regulated using 0.1 M NaOH or 0.1 M HCl, and monitoring was conducted with a pH meter (PHS-25). Apart from the experiments investigating the pH effect, all tests were carried out under an initial pH.

*Analytical methods.* The removal efficiency of AO7 was determined by the following equation:

$$\mathrm{AO}7\;\mathrm{removal}\;(\%)\;=\;\frac{C_{0}-C_{t}}{C_{0}}\times 100\%$$

where *C*_0_ and *C_t_* are the AO7 concentration at 0 and t moment in wastewater of catalytic degradation. The AO7 concentration was quantified by UV-Vis spectrophotometry at its characteristic wavelength of 486 nm.

## 3. Results and Discussion

**Figure 1:**
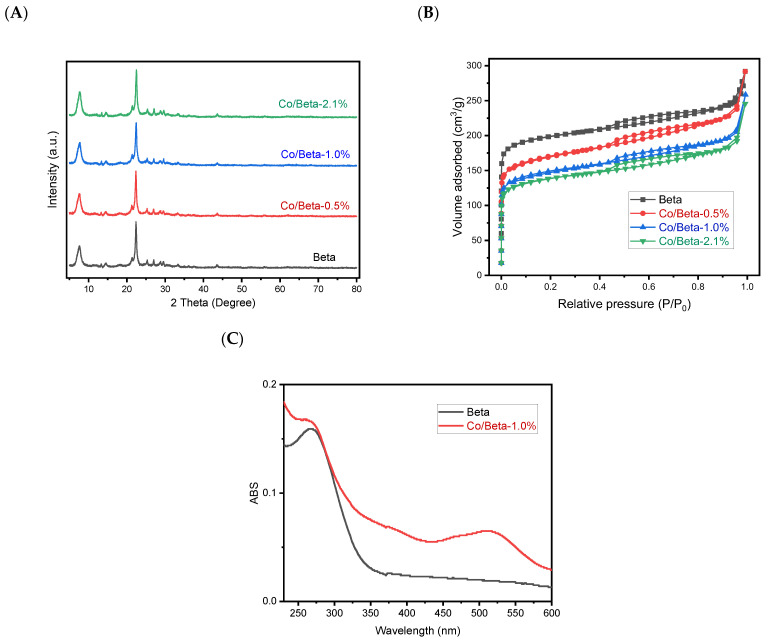
(**A**) XRD patterns, (**B**) N_2_ adsorption isotherms, and (**C**) UV-Vis spectroscopy of Beta and Co/Beta-1.0% catalysts.

**Figure 2:**
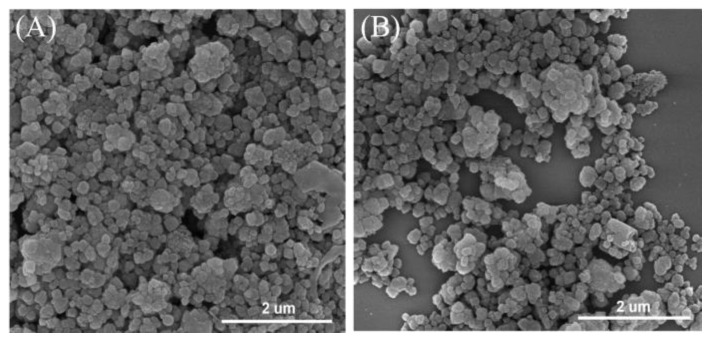
(**A**,**B**) SEM images of Co/Beta-1.0%.

**Figure 3:**
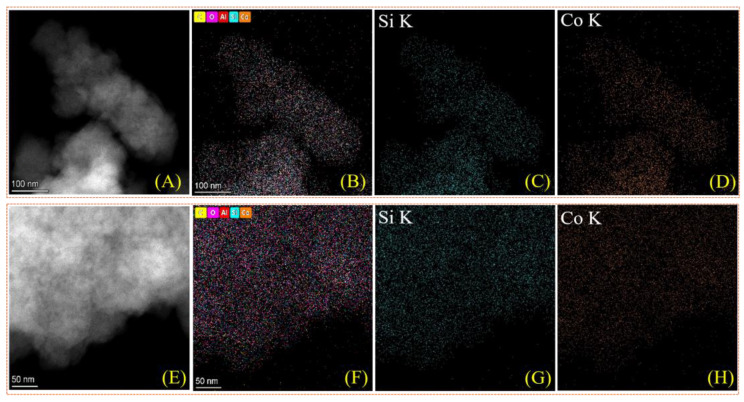
HAADF-STEM images (**A**,**E**) and elemental mapping (**B**–**D**) for (**A**) image and (**F**–**H**) for (**E**) image of Co/Beta-1.0%.

Figure 1A provides the XRD patterns corresponding to the Beta and Co/Beta samples, which showed typical diffractions assigned to the *BEA structure (PDF#47-0183). Notably, the XRD patterns of both samples are very similar, and the diffractions of metallic cobalt or cobalt oxide were undetectable on the Co/Beta sample, confirming the uniform distribution of cobalt species. The N_2_ sorption technique was employed to characterize the porosity of the samples. As illustrated in Figure 1B, the adsorption and desorption isotherms display a hysteresis loop at high pressure characteristic of a type IV isotherm, suggesting that the inherent microporous structure of Beta zeolite remains intact. The BET surface area and pore volume of Co/Beta samples showed a slight reduction relative to Beta zeolite, attributable to the addition of cobalt species. The surface areas and pore volumes for these materials are shown in Table 1. Based on the measured Co content of 2.1 wt% and the framework Si/Al ratio of 15, we estimate the Co^2+^ exchange degree to be about 65%. This value was calculated assuming that each Co^2+^ ion replaces two framework Al sites, which is consistent with the charge-balancing requirement in zeolite ion exchange. The theoretical maximum Co loading was derived from the total Al content in the framework, and the actual Co^2+^ molar amount was obtained from the elemental analysis. These data confirm the well-maintained *BEA structure after loading cobalt species, which is further confirmed by the SEM and TEM characterization. Figure 2 shows that the crystal morphology of zeolite remains unchanged after cobalt loading. The HAADF-STEM mapping images further confirm the uniform distribution of cobalt species, indicating the Co atoms were not agglomerated during the synthesis process (Figure 3).

UV-Vis spectroscopy is extensively utilized for analyzing the oxidation states and local coordination structures of cobalt centers within Co-incorporated zeolite frameworks [41]. Compared with Beta zeolite, the Co/Beta sample shows a distinct broad band in the visible range (490–540 nm), as shown in Figure 1C, which can be attributed to the presence of Co^2+^ species in the zeolite framework. This feature is in good agreement in association with the metal species embedded in the zeolite prepared by the ion-exchanged method [42].

**Figure 4:**
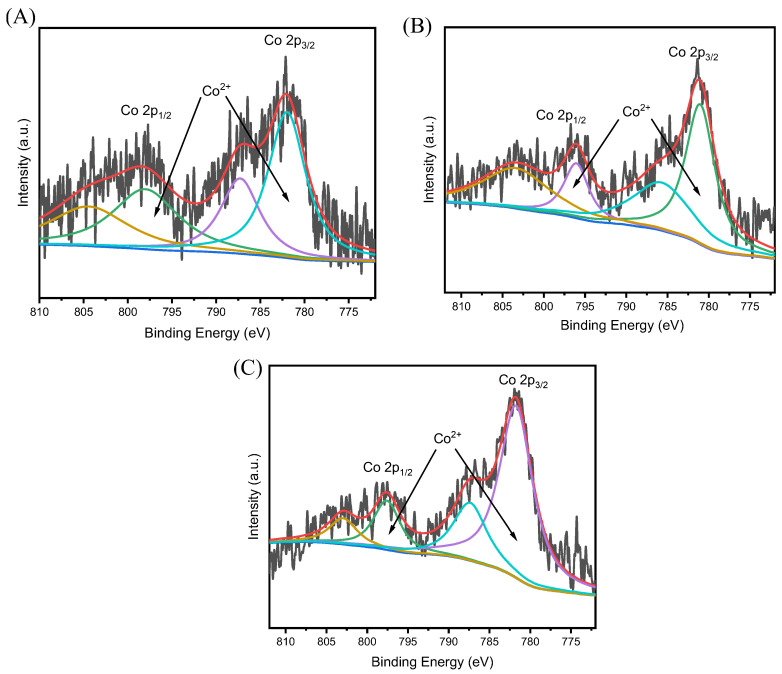
XPS patterns of different samples (**A**) Co/Beta-0.5%, (**B**) Co/Beta-1.0% and (**C**) Co/Beta-2.1%.

X-ray photoelectron spectroscopy (XPS) is a widely used technique for detailed identification of cobalt species. Figure 4 presents the XPS spectra of three Co/Beta samples. In the Co 2p region for all Co/Beta samples, prominent doublet peaks are observed and accompanied by satellite peaks at higher binding energies. Specifically, the peaks at 781.9 eV (Co 2p_3/2_) and 798.0 eV (Co 2p_1/2_) correspond to Co^2+^ species in the Co/Beta-0.5% sample (Figure 4A). For the Co/Beta-1.0% catalyst (Figure 4B), peaks observed at binding energies of 781.2 eV (Co 2p_3/2_) and 797.0 eV (Co 2p_1/2_) are assigned to Co^2+^. In the XPS spectrum of Co/Beta-2.1% (Figure 4C), Co^2+^ species are identified via characteristic peaks centered at binding energies of 781.8 eV (Co 2p_3/2_) and 797.7 eV (Co 2p_1/2_). These analysis results indicate that Co species mainly exist in the molecular sieve skeleton in the form of ion-exchanged Co^2+^.

**Figure 5:**
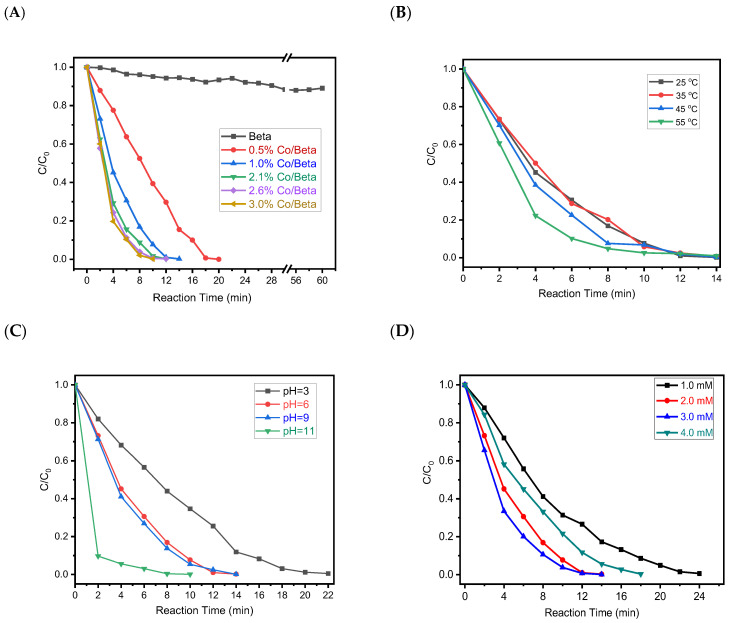
(**A**) Catalytic degradation of AO7 for catalysts with different cobalt contents (reaction conditions: 30 mL of a 0.2 mM solution of AO7, 2.0 mM of PMS, 10 mg of catalysts, initial pH, 25 ± 2 °C); study on the influencing factors of catalytic performance for Co/Beta-1.0%, including the following content, (**B**) reaction temperature (reaction conditions: 30 mL of a 0.2 mM solution of AO7, 2.0 mM of PMS, 10 mg of catalyst, initial pH), (**C**) pH effect of solution (reaction conditions: 30 mL of a 0.2 mM solution of AO7, 2.0 mM of PMS, 10 mg of catalyst, 25 ± 2 °C), and (**D**) PMS loading effect (reaction conditions: 30 mL of a 0.2 mM solution of AO7, 10 mg of catalyst, initial pH, 25 ± 2 °C).

The catalytic activity of Co-based catalysts was examined by the degradation of AO7 in water solvent, as shown in Figure 5. Firstly, considering the generally known excellent adsorption capacity of the molecular sieve, we evaluated the adsorption performance of Beta zeolite for AO7. As shown in Figure 5A, the physical adsorption for AO7 on the Beta zeolite is slight and negligible. At low Co loadings, the apparent catalytic activity was limited, likely due to insufficient active-site density at the lower Co content. As the Co content increased, the performance improved progressively. In contrast, more than 99.5% removal efficiency can be achieved in 10 min by the Co/Beta/PMS system in the reaction as the Co content gradually increases, much higher than most of the catalysts shown in Table 2, demonstrating the superior catalytic performance of Co/Beta for AO7 degradation. However, when the Co loading exceeded 2.1 wt%, no significant enhancement in activity was observed. Therefore, we selected the three samples with less than 2.1 wt% as the representative loading in this study. This choice reflects a balance between catalytic efficiency and cobalt utilization. Comparative studies were performed using Beta, ZSM-5, and Y zeolites, three of the most widely employed catalytic materials in industrial applications. Among these, Co/Beta zeolite exhibited the most favorable catalytic performance under the reaction conditions (Table 2). As the molecular size of AO7 significantly exceeds the micropore diameter of Beta zeolite, its direct adsorption within the zeolite channels is expected to be very limited. Therefore, the primary role of the zeolite may not be substrate adsorption, but rather to stabilize and disperse Co^2+^ species effectively. This high dispersion facilitates the activation of PMS and the generation of reactive radicals, which subsequently degrade AO7 in the bulk solution.

The degradation performance of AO7 on Co/Beta catalysts was systematically determined by three critical parameters: PMS concentration, reaction temperature, and solution pH, which collectively dictate the PMS activation efficiency. Figure 5B–D displays the impact of the various reaction conditions on AO7 removal. A gradual enhancement in removal rate was observed when the temperature was elevated from 25 °C to 45 °C (Figure 5B). A possible explanation is that the elevated temperatures can promote electron transfer between Co/Beta and PMS, resulting in an increase in the generation of reactive oxygen species (ROS) and thereby enhancing the catalytic activity for AO7 removal. The pH value of the solution is considered one of the important parameters affecting the AOP reaction process. As illustrated in Figure 5C, the Co/Beta/PMS system exhibited a slight increase in AO7 removal efficiency as the solution pH increased from 6.0 to 9.0. The system is more sensitive to alkaline conditions. A significant enhancement in catalytic efficiency is observed at pH 11. However, conditions of strong acidity (pH = 3.0) were relatively adverse to the degradation process. The reason may be that under strong acidic conditions, the active sites on the catalyst surface are excessively occupied by hydrogen ions, which hinders the adsorption and activation of PMS and leads to a decrease in the efficiency of free radical generation. Figure 5D shows that the performance of the Co/Beta/PMS system was considerably dependent on the PMS concentration. Raising the PMS concentration from 1.0 to 3.0 mM resulted in improved degradation efficiency, presumably due to the increase in the formation of reactive species, accelerating the degradation of AO7. Conversely, a marginal decrease in degradation efficiency occurred when the PMS concentration was raised beyond this range to 4.0 mM, primarily caused by the self-quenching effect along with reduced production of reactive oxygen species [43,44]. Moreover, ICP-OES analysis was performed after reaction and separation of the solid materials; the cobalt concentration in the liquor was lower than 5 ppb, and the cobalt loading remained consistent within experimental error, indicating negligible leaching and good structural stability of the catalyst throughout the process.

**Table 2:** The removal efficiency of various catalysts for AO7 degradation.

Catalysts	C_AO7_ (mM)	C_PMS_ (mM)	C_Catalyst_ (g·L^−1^)	t (min)	Removal Efficiency (%) *^b^*	References
CUST-562	0.06	0.16 g/L *^a^*	0.12	30	93.5	[45]
HDCo@C-800	0.03	0.76 g/L *^a^*	0.1	60	>99.5	[46]
CoCuAl-LDOs	0.06	0.1 g/L *^a^*	0.1	30	>99.5	[47]
Co-HPNC	0.1	1.0	0.05	10	98.1	[48]
Co-Fc-MOFs	0.06	4.0	0.25	90	83.7	[49]
Co-CoO@BC	0.06	1.0	0.06	20	95.0	[50]
Co–MIL-101(Fe)	0.1	8.0	0.2	180	98.0	[51]
Co/SBA-15	0.2	5.0	0.5	90	>99.5	[52]
Co_3_O_4_/NF	0.1	0.5	2.0 mM *^a^*	30	>99.5	[53]
nano-Co_3_O_4_	0.2	2.0	0.5	30	>99.5	[54]
Co/ZSM-5	0.2	2.0	0.3	16	>99.5	Pw *^c^*
Co/Y	0.2	2.0	0.3	14	>99.5	Pw *^c^*
Co/Beta	0.2	2.0	0.3	10	>99.5	Pw *^c^*

*^a^* Based on the suffix unit. *^b^* Reaction temperature 25 ± 2 °C. *^c^* Present work.

**Figure 6:**
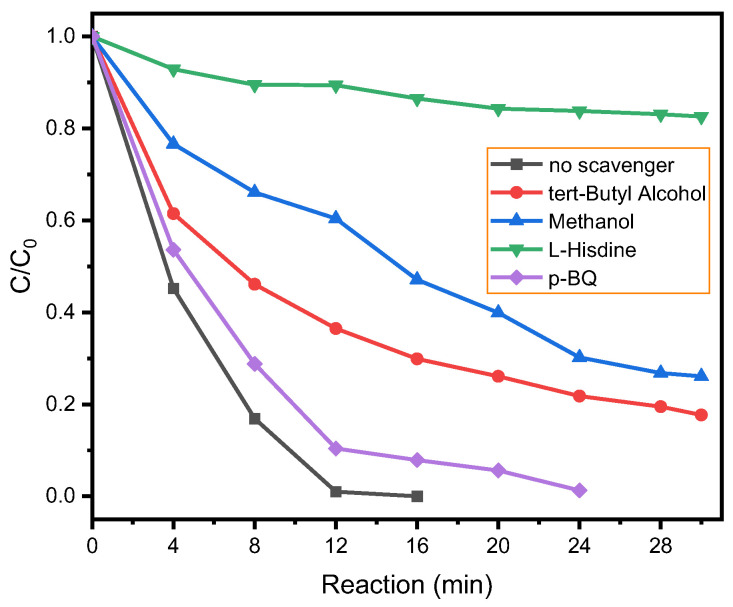
Radical quenching experiments with several scavenging reagents on the catalytic degradation of AO7 in the Co/Beta/PMS system. (Reaction conditions: 30 mL of a 0.2 mM solution of AO7, 2.0 mM of PMS, 10 mg of catalysts, 120 mM of L-histidine or p-BQ, 200 mM of methanol or tert-butyl alcohol, initial pH, 25 ± 2 °C).

Typically, in PMS-based advanced oxidation processes, the removal of organic contaminants is largely ascribed to reactive oxygen species such as SO_4_^•−^, •OH, HO_2_•/•O_2_^−^, and singlet oxygen (^1^O_2_). The involvement of ROS in the Co/Beta system was investigated through radical-scavenging experiments employing various quenchers: methanol targeting •OH and SO_4_^•−^, p-benzoquinone (p-BQ) for •O_2_^−^, and L-histidine for ^1^O_2_. As illustrated in Figure 6, the addition of p-BQ caused only a slight inhibition of AO7 degradation, indicating a minor role of •O_2_^−^ in the process. The addition of methanol led to a 74% reduction in degradation efficiency. On the other hand, introducing L-histidine caused a notable suppression of degradation. Even after 30 min, less than 20% of AO7 had been removed. In summary, we can draw the conclusion that the inhibition of degradation efficiency followed the ascending order L-histidine > methanol > p-BQ, which indicates that singlet oxygen (^1^O_2_) is the key reactive species in the reaction system.

**Figure 7:**
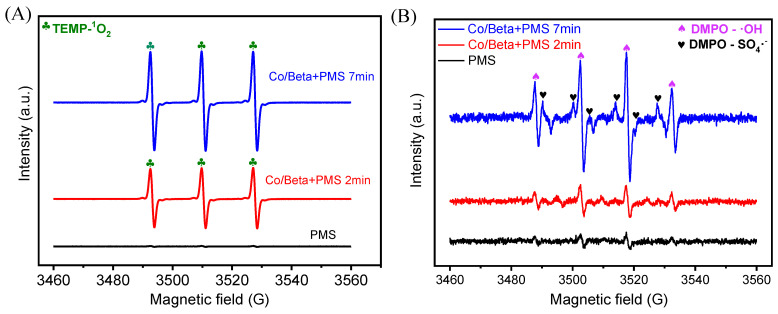
EPR trapping signals of TEMP-^1^O_2_ (**A**), DMPO- SO_4_^•−^ and DMPO-•OH (**B**). Conditions for EPR tests: 0.2 g·L^−1^ of Co/Beta-1.0%, 2.0 mM of PMS, 1.0 mM of TEMP or DMPO.

For additional validation of the involvement of the oxygen species mentioned above, EPR spectroscopy was applied with the DMPO and TEMP employed as spin-trapping agents during the reaction. The determination characterization of singlet oxygen ^1^O_2_ was shown in Figure 7A. It is shown that when there is only PMS, almost no characteristic peak signal of ^1^O_2_ is generated. When the Co/Beta catalyst was added and the reaction time was two min, the obvious ^1^O_2_ characteristic triple peak (1:1:1) appeared. The reaction was continued to seven min before measurement, and it was found that the signal of the characteristic peak was more intense, directly indicating that singlet oxygen ^1^O_2_ played a very key role in the catalytic degradation of AO7 in the Co/Beta catalytic system, consistent with the radical quenching experiments above.

**Figure 8:**
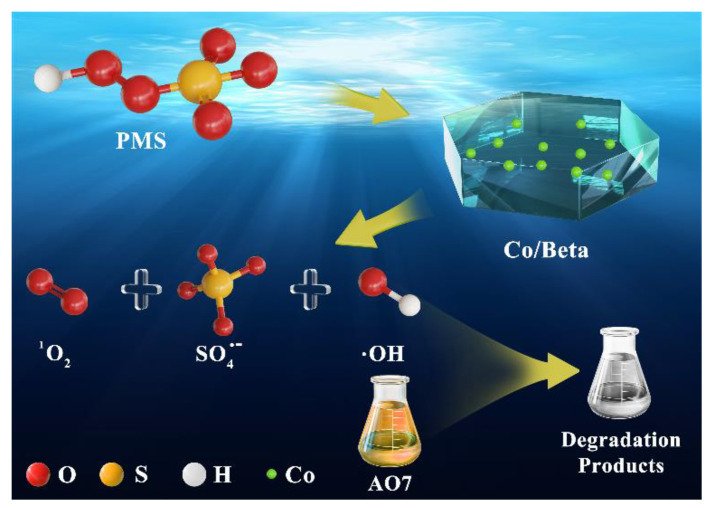
A proposed catalytic mechanism for AO7 degradation.

The EPR capture test of SO_4_^•−^ and •OH is shown in Figure 7B. It can be seen that when only PMS is available, very weak signal peaks are generated. When Co/Beta catalyst is added and measured after two min of reaction, obvious characteristic peaks of SO_4_^•−^ and •OH (1:2:2:1) appear. With the progress of the reaction, the peak signal became more intense, measured at seven min, suggesting that both SO_4_^•−^ and •OH radicals participated in the catalytic degradation and contributed to the reaction. However, no signal corresponding to DMPO- •O_2_^−^ was observed. This phenomenon may be explained by the interaction between •O_2_^−^ and •OH, leading to the formation of singlet oxygen (^1^O_2_). As confirmed by the free radical quenching and detection experiments above, we can draw the conclusion that singlet oxygen is the most important active species in the AO7 catalytic degradation reaction (Figure 8). In addition, SO_4_^•−^ and •OH oxygen species also play a very important role in the excellent degradation performance.

## 4. Conclusions

In this work, we demonstrate that cobalt can be effectively introduced into the Beta zeolite framework via a simple ion-exchange method, resulting in highly active catalysts for PMS activation and pollutant degradation in water. Compared to conventional Co-based catalysts or Co/MOFs, the Co/Beta catalysts exhibit superior catalytic performance, lower potential cost, and greater feasibility for scale-up. Moreover, the purely inorganic nature of Co/Beta zeolites ensures structural robustness under radical-rich reaction conditions. Given that zeolites such as Beta are already widely used in the petrochemical industry, our strategy of extending their application to PMS activation through cobalt incorporation offers a practical and scalable pathway to develop efficient environmental catalysts with direct application potential.

## Figures and Tables

**Table 1:** Physical parameters of the Beta and Co/Beta samples.

Catalysts	Co(wt%) *^a^*	Surface Area (m^2^/g) *^b^*	Pore Volume (cm^3^/g) *^c^*
Beta	0	554	0.23
Co/Beta-0.5%	0.5	545	0.22
Co/Beta-1.0%	1.0	524	0.22
Co/Beta-2.1%	2.1	475	0.20

*^a^* Results obtained from ICP-OES. *^b^* Results obtained from the BET Surface area. *^c^* Results obtained from BJH Adsorption cumulative volume of pores.

## Data Availability

Data is contained within the article.
